# Association of Depressive Symptom Trajectory With Physical Activity Collected by mHealth Devices in the Electronic Framingham Heart Study: Cohort Study

**DOI:** 10.2196/44529

**Published:** 2023-07-14

**Authors:** Xuzhi Wang, Chathurangi H Pathiravasan, Yuankai Zhang, Ludovic Trinquart, Belinda Borrelli, Nicole L Spartano, Honghuang Lin, Christopher Nowak, Vik Kheterpal, Emelia J Benjamin, David D McManus, Joanne M Murabito, Chunyu Liu

**Affiliations:** 1 Department of Biostatistics Boston University School of Public Health Boston, MA United States; 2 Tufts Clinical and Translational Science Institute Tufts University Boston, MA United States; 3 Institute for Clinical Research and Health Policy Studies Tufts Medical Center Boston, MA United States; 4 Center for Behavioral Science Research Boston University Henry M Goldman School of Dental Medicine Boston, MA United States; 5 Section of Endocrinology, Diabetes, Nutrition, and Weight Management Boston University Chobanian and Avedisian School of Medicine Boston, MA United States; 6 Department of Medicine University of Massachusetts Chan Medical School Worcester, MA United States; 7 Care Evolution Ann Arbor, MI United States; 8 Section of Preventive Medicine and Epidemiology and Cardiovascular Medicine, Department of Medicine Boston University Chobanian and Avedisian School of Medicine Boston, MA United States; 9 Framingham Heart Study, Boston University and National Heart, Lung, and Blood Institute Framingham, MA United States; 10 Department of Epidemiology Boston University School of Public Health Boston, MA United States; 11 Cardiology Division, Department of Medicine University of Massachusetts Chan Medical School Worcester, MA United States; 12 Department of Quantitative Health Sciences University of Massachusetts Chan Medical School Worcester, MA United States; 13 Section of General Internal Medicine, Department of Medicine Boston University Chobanian and Avedisian School of Medicine Boston, MA United States

**Keywords:** depression, mobile health, risk factors, physical activity, eCohort, Framingham Heart Study

## Abstract

**Background:**

Few studies have examined the association between depressive symptom trajectories and physical activity collected by mobile health (mHealth) devices.

**Objective:**

We aimed to investigate if antecedent depressive symptom trajectories predict subsequent physical activity among participants in the electronic Framingham Heart Study (eFHS).

**Methods:**

We performed group-based multi-trajectory modeling to construct depressive symptom trajectory groups using both depressive symptoms (Center for Epidemiological Studies-Depression [CES-D] scores) and antidepressant medication use in eFHS participants who attended 3 Framingham Heart Study research exams over 14 years. At the third exam, eFHS participants were instructed to use a smartphone app for submitting physical activity index (PAI) surveys. In addition, they were provided with a study smartwatch to track their daily step counts. We performed linear mixed models to examine the association between depressive symptom trajectories and physical activity including app-based PAI and smartwatch-collected step counts over a 1-year follow-up adjusting for age, sex, wear hour, BMI, smoking status, and other health variables.

**Results:**

We identified 3 depressive symptom trajectory groups from 722 eFHS participants (mean age 53, SD 8.5 years; n=432, 60% women). The low symptom group (n=570; mean follow-up 287, SD 109 days) consisted of participants with consistently low CES-D scores, and a small proportion reported antidepressant use. The moderate symptom group (n=71; mean follow-up 280, SD 118 days) included participants with intermediate CES-D scores, who showed the highest and increasing likelihood of reporting antidepressant use across 3 exams. The high symptom group (n=81; mean follow-up 252, SD 116 days) comprised participants with the highest CES-D scores, and the proportion of antidepressant use fell between the other 2 groups. Compared to the low symptom group, the high symptom group had decreased PAI (mean difference –1.09, 95% CI –2.16 to –0.01) and the moderate symptom group walked fewer daily steps (823 fewer, 95% CI –1421 to –226) during the 1-year follow-up.

**Conclusions:**

Antecedent depressive symptoms or antidepressant medication use was associated with lower subsequent physical activity collected by mHealth devices in eFHS. Future investigation of interventions to improve mood including via mHealth technologies to help promote people’s daily physical activity is needed.

## Introduction

Depression is described as having negative feelings such as sadness or a loss of interest in activities that a person once enjoyed. Depression is a common and serious medical illness that affects millions of Americans each year [1-3]. A recent study reported that the prevalence of elevated depressive symptoms is 32.8% in a national, population-representative, longitudinal study of US adults in 2021 [4]. Depression and physical activity have bidirectional relationships. Physical inactivity is a risk factor for depression [5-7]. Evidence suggests that the use of behavioral therapies such as exercise can help improve the treatment of depression [8]. Low doses of physical activity are protective against depression [5], and regular exercise can result in substantial reductions in depression in the general older adult population [9].

Most previous studies evaluated the relationships of depression with subjectively measured physical activity (ie, self-reported physical activity). Few studies have investigated how variation in mood is associated with physical activity [10-12]. An investigation of depressive symptoms and self-reported physical activity in the 1958 British Birth Cohort in adults from age 23 to 50 years suggests that depressive symptoms in early adulthood may be a barrier to physical activity [11]. The Whitehall Study reported that participants with depressive symptoms at baseline were more likely to fail to meet the recommended levels of physical activity at follow-up [12]. The Coronary Artery Risk Development in Young Adults study identified a longitudinal association between baseline depressive symptoms and self-reported moderate to vigorous intensity physical activity over 10-year follow-up but not with objectively measured moderate to vigorous intensity physical activity [13]. To our best knowledge, no study considered the association between the longitudinal trajectory of depressive symptoms and physical activity measured with mobile devices both self-reported and objectively.

Mobile health (mHealth) devices, including smartphones and wearables, are increasingly used by many Americans to monitor their health and health-related behaviors [14]. The electronic Framingham Heart Study (eFHS) is an e-cohort nested within the larger multigenerational Framingham Heart Study (FHS). The participants in the eFHS were instructed to use a smartphone app to submit their health survey data. They were also invited to wear a study smartwatch to record daily step counts. The primary aim of this investigation was to examine the association of antecedent depressive symptoms and antidepressant medication use with subsequent physical activity collected during eFHS. We hypothesized that the long-term trend of depressive symptoms and antidepressant use prior to enrollment in the eFHS was associated with subsequent lower levels of physical activity during eFHS.

## Methods

### Overview

**Figure 1:**
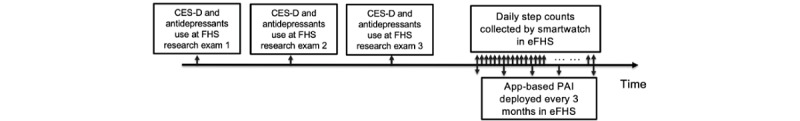
The timeline of data collection for CES-D scores and antidepressant use at Framingham Heart Study (FHS) research exams, as well as app-based PAI and smartwatch-collected step. CES-D: Center for Epidemiological Studies-Depression; eFHS: electronic Framingham Heart Study; PAI: physical activity index.

We captured the longitudinal trend of depressive symptoms and categorized them into distinct trajectory groups based on a composite depressive symptom score and the use of antidepressant medications assessed at FHS research examinations 1-3 across 14 years prior to the eFHS. We assessed whether the constructed depressive symptom patterns predicted the average level of physical activity collected by mHealth devices during short-term follow-up in the eFHS. Last, we examined whether the constructed depressive symptom patterns predicted habitual physical activity within an intermediate time span of 1-year follow-up in the eFHS (Figure 1). Study method and results are reported following the STROBE (Strengthening the Reporting of Observational Studies in Epidemiology) statement for cross-sectional studies.

### Study Participants

**Figure 2:**
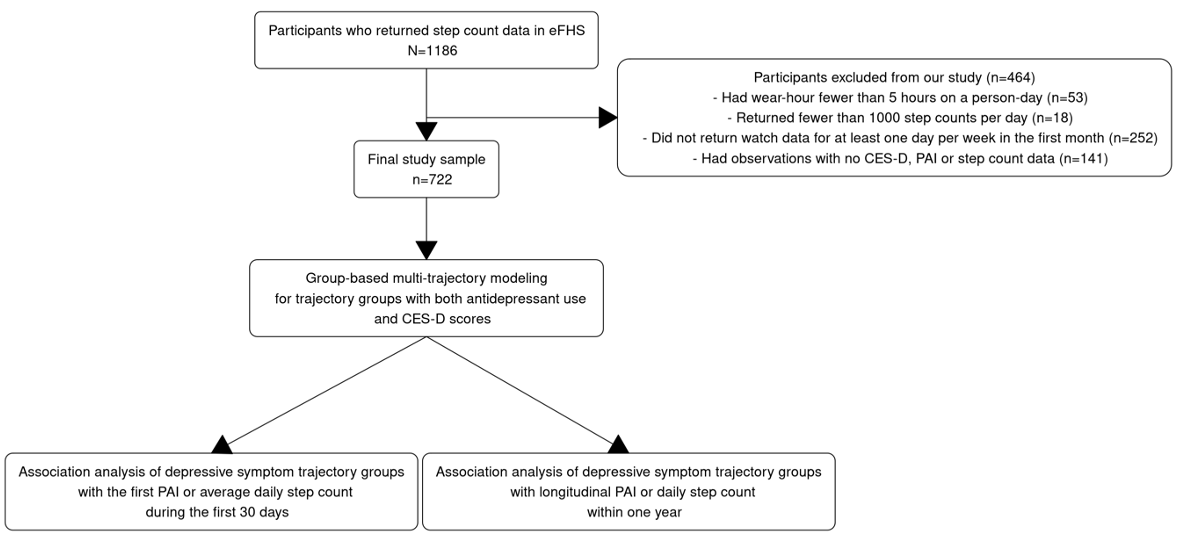
Study sample selection and statistical analyses. CES-D: Center for Epidemiological Studies-Depression; eFHS: electronic Framingham Heart Study; PAI: physical activity index.

The FHS began in 1948 by enrolling 5209 residents (original cohort) from Framingham, Massachusetts. In 1971, a total of 5214 participants who were offspring of the original cohort, and the spouses of the offspring participants were enrolled in an offspring cohort. During 2002 and 2005, the FHS enrolled the third generation (Gen 3) cohort (n=4094), which included grandchildren of the original cohort. During the same time, the FHS enrolled 410 residents who were from underrepresented racial or ethnic groups in or near Framingham (Omni2), and New offspring spouse cohort (NOS, n=103). This study included participants in the Gen 3, Omni2, and NOS cohorts who have undergone 3 research examinations every 6 to 8 years (exam 1: 2002-2005, exam 2: 2008-2011, exam 3: 2016-2019) [15]. At research exam 3 (2016 to 2019), English-speaking participants who owned a smartphone (including iPhone 4S [Apple Inc] or higher with iOS version 8.2 [Apple Inc] or higher or beginning October 30, 2017, an Android phone) were invited to enroll in the eFHS [16]. Most participants who agreed to participate in eFHS downloaded the eFHS smartphone app from the Apple App Store at the in-person research center visit with help from the FHS-trained staff. Some participants chose to register in eFHS after leaving the research center with written instructions provided by eFHS staff. The sample size of our main analysis included participants who attended FHS examinations 1-3 and had self-reported physical activity and valid Apple Watch step data (defined below) for the first month following their third examination (Figure 2).

### Ethics Approval

All study participants provided informed consent for the study. The protocols for eFHS and the FHS were approved by the Institutional Review Board (H-36586 and H-32132) at the Boston University Medical Center.

### Center for Epidemiological Studies-Depression Score and Antidepressant Medications for Trajectory Building

We selected participants from Gen 3, OMNI2, and NOS cohorts who participated in all 3 FHS exams. Trained technicians administered the 20-item Center for Epidemiological Studies-Depression (CES-D) scale at each of the 3 research exams. The CES-D scale assesses how often individuals experienced depressive symptoms over the past week, with the total score ranging from 0 to 60. Each item has a score that ranges from 0 to 3 [17]. In 16 of the 20 CES-D questions, a higher score corresponds to a higher frequency of depressive symptoms. For the remaining 4 questions, a higher score corresponds to a lower frequency of depressive symptoms. We reversed the scores of these 4 questions and summed up the individual scores over the 20 questions to construct the composite CES-D score for each participant at every attended exam. A threshold of 16 or greater is commonly used to identify individuals who have depressive symptoms [17].

At each FHS examination, participants were asked to bring all medications to the research center in a medication bag. All antidepressant medications were recorded, and we identified antidepressant medications such as sertraline, fluoxetine, and citalopram. We considered that participants used antidepressant medication if any antidepressant medication was identified at a given FHS exam.

### Assessment of Step Counts and Physical Activity Index

At exam 3, iPhone users were provided with the choice to participate in eFHS or not to participate. For users who chose to participate in the study, they had a choice to use the study smartwatch, an Apple Watch Series 0 (Apple Inc), or the first generation of Apple Watch. eFHS study staff assisted participants with pairing the watch to their smartphone in the Research Center. Participants were asked to wear the watch daily for at least 1 year [16].

We defined a person-day as a day for which a participant returned any amount of smartwatch data, and we defined “wear hours” as the number of hours during which a participant wore the smartwatch on a person-day. We excluded person-days for which participants had fewer than 5 wear hours (Figure 2) [18]. To minimize inaccurate data points, we further excluded person-days for which participants had less than 1000 step counts per day [19]. We then removed participants who failed to return watch data for at least 1 day per week in the first month after the participant started returning data in eFHS, because we planned to examine the association between antecedent depressive symptoms and average step counts during the first 30 days. In addition, we used data collected from the eFHS enrollment at the exam 3 to 1-year follow-up because one of the aims of this study was to investigate the association between antecedent depressive symptoms with habitual physical activity over an intermediate time span.

Daily step counts were collected by the smartwatch. Baseline average daily step counts were calculated as the mean of the daily step counts during the first 30 days after a participant’s first return of the step data. Longitudinal step counts referred to the repeated daily step counts within the 1-year follow-up since the first return of the step data in eFHS.

In addition to the physical activity data that were objectively measured from the smartwatch, we also assessed the self-reported physical activity index (PAI) collected by the eFHS smartphone app for comparison. The eFHS smartphone app included a survey to assess physical activity using the Framingham PAI. The physical activity survey was sent through the app every 3 months for 1 year. The PAI was calculated as a composite score based on the number of hours spent sleeping, or in sedentary, slight, moderate, and heavy activities during a 24-hour period. We assigned weights of 1, 1.1, 1.5, 2.4, and 5 to sleep, sedentary, slight, moderate, and heavy activity, respectively [20-22]. Baseline PAI was defined as the first app-based PAI record collected within the first 3 months by the eFHS smartphone app. Longitudinal PAI referred to the repeated app-based PAI collected within 1-year follow-up since the baseline PAI.

Figure 1 displays the timeline of data collection for CES-D scores and antidepressant medication use at FHS research exams, as well as for app-based PAI and smartwatch-collected step counts.

### Covariates

Demographic variables were measured during exam 3 at the research center. BMI was calculated by dividing body weight (kg) by height (meters) square. Obesity was defined as a BMI value of 30 kg/m^2^ or higher. Using the highest education level the participant achieved at exam 3, we defined the education variable into 3 categories: “less than high school,” “completed high school or some college,” and “bachelor’s degree or higher.” Marital status was defined into 5 categories: “divorced,” “married,” “never married,” “separated,” and “widowed.” Lipid-lowering treatment was defined as a self-report of receiving lipid treatment in the year before the exam. Hypertension was defined by systolic blood pressure of 130 mm Hg or higher, or diastolic blood pressure of 80 mm Hg or higher, or antihypertensive medication use. We also defined current smoking as participants who reported smoking at least 1 cigarette per day in the year before the exam. Several laboratory variables, including total cholesterol, high-density lipoprotein cholesterol, triglycerides, and fasting blood glucose, were also measured at exam 3. Diabetes was defined as the prior diagnosis, use of diabetes medications, hemoglobin A_1c_ (HbA_1c_) ≥6.5%, fasting blood glucose ≥126 mg/dL, or random plasma glucose ≥200 mg/dL; prevalent cardiovascular disease (CVD) was defined as history of heart failure, myocardial infarction, angina, stroke, or intermittent claudication and coded.

### Statistical Analyses

#### Overview

We first examined the main characteristics of the participants and compared the characteristics between those who remained in our study and those who were excluded. Next, we built the trajectory groups based on the longitudinal patterns of depressive symptoms (CES-D scores) and antidepressant medication use. Furthermore, we performed association analyses to examine the relationships between trajectory groups and physical activity variables during the first 30 days and during the 1-year follow-up.

#### Trajectory Modeling

We constructed the depressive symptom trajectories across exam 1 (2002-2005) to exam 3 (2016-2019) to capture different temporal patterns of CES-D scores and antidepressant medication use. We used group-based multi-trajectory [23] modeling to construct the trajectory patterns with both the antidepressants use and the continuous CES-D scores from research exams 1 to 3 (occurring over the 14 years before the eFHS). The trajectories of CES-D scores and antidepressant use were specified as a function of exam, adjusted for baseline age and sex. The group-based multi-trajectory modeling is an extension of univariate group-based trajectory modeling. The group-based multi-trajectory modeling identifies latent subgroups of individuals following similar longitudinal trajectories across multiple correlated outcome variables over time. This method is flexible to model the interrelationship of multiple clinically relevant variables. Group-based multi-trajectory models with different trajectory shapes (ie, linear or quadratic) and with a different number of groups (ranging from 1 to 4) were tested. To determine the optimal number of trajectory groups, we started with a single group, and added 1 more group at a time. We used the Bayesian information criterion statistic to evaluate the model fit with different groups and shapes, with an additional requirement that each group consists of at least 5% of the sample [24].

#### Association Analysis of Depressive Symptom Trajectory Groups With the First App-Based PAI of eFHS or the Average Daily Step Counts During the First 30 Days of eFHS

We investigated both self-reported PAI and objectively measured step count in 3 trajectory groups. First, we performed a correlation analysis for the first app-based PAI and the average daily step count during the first 30 days. Next, we conducted analysis of covariance models to compare the first app-based PAI and the daily average step counts during the first 30 days (outcome) in the depressive symptom trajectory groups (predictor). Models were adjusted for age, sex (additionally adjusted for average daily wear hour for steps; model 1) and further adjusted for BMI, smoking status, prevalent CVD, current diabetes status, education, marital status, hypertension status, fasting blood glucose, high-density lipoprotein cholesterol, total cholesterol, triglycerides, and lipid treatment at exam 3 as additional variables to those in model 1 (model 2).

#### Association Analysis of the Depressive Symptom Trajectory Groups With the Longitudinal PAI or Daily Step Counts

We further examined the associations of the depressive symptom trajectory groups as the independent variable with the longitudinal PAI and daily step counts collected over a 1-year follow-up in the eFHS. We used a linear mixed model with longitudinal PAI values collected every 3 months as the outcome, adjusting for age, sex, and the number of days between baseline and a survey collection. We further adjusted for all the other covariates in model 2 in the previous section. We used linear mixed models with the repeated daily step counts as the outcome, adjusting for age, sex, the number of days between baseline and data collection, and the daily wear hour of the smartwatch use. Similarly, the model with daily step counts was further adjusted for other covariates.

All statistical analyses were conducted using SAS (version 9.3; SAS Institute) and R (version 4.0; R Foundation for Statistical Computing). Group-based multi-trajectory modeling was performed using the SAS Proc Traj [23]. We used 2-tailed *P*<.05 for significance.

## Results

### Participant Characteristics

A total of 3196 FHS participants (2873 from Gen 3, 49 from NOS, and 274 from Omni2) attended research exams 1 to 3. Of these 3196 participants, 2150 participants enrolled in the eFHS study, and 1186 participants returned app-based PAI and smartwatch-collected step counts data [25]. After applying 3 exclusion criteria to those who had both PAI and step counts records and removing those with missing values in any of the covariates, 722 individuals (median follow-up 14 years; mean age 53 years, SD 8.5 years; n=432, 60% women; n=656, 91% White) were included in the association analyses (Figure 2). Within a 1-year window, the participants had a median follow-up time of 52 weeks (Q1-Q3=50, 52). Compared to FHS participants who declined enrollment in eFHS, our study sample was significantly younger (mean 53, SD 8.5 years vs mean 57, SD 10 years; *P*<.001), had a higher proportion of women (432/722, 60% vs 632/1275, 50%; *P*<.001), lower CES-D scores (mean 5.79, SD 6.63 vs mean 7.27, SD 7.47; *P*<.001), lower BMI (mean 28.2, SD 5.6 vs mean 28.9, SD 6; *P*=.04), and a higher proportion of college graduates (495/722, 69% vs 605/1275, 47%; *P*<.001). Compared to other eFHS participants who accepted enrollment but did not meet the inclusion criteria, our study sample was significantly more likely to be women (432/722, 60% vs 670/1255, 53%; *P*=.006) and had a higher education level (bachelor’s degree or higher 495/722, 69% vs 802/1255, 64%; *P*=.04; Multimedia Appendix 1).

### Depressive Symptom Trajectory Groups

**Figure 3:**
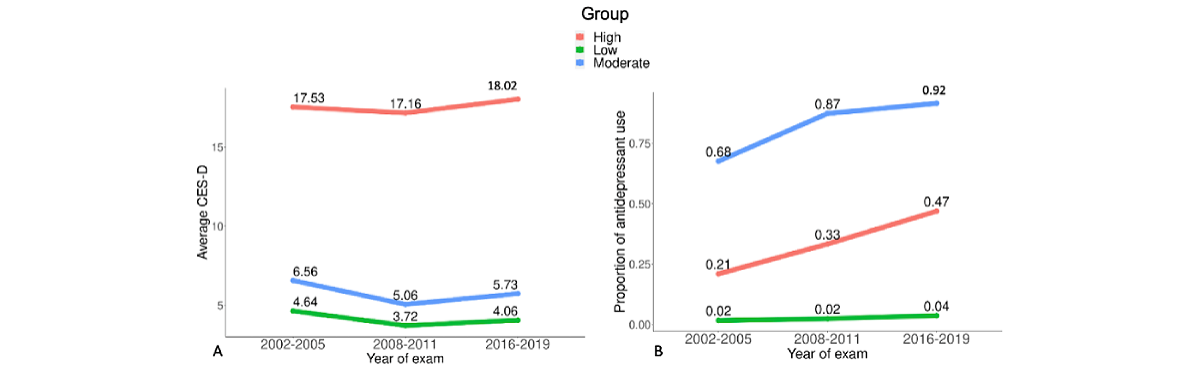
The relationship of trajectory groups with CES-D scores and antidepressant medication use. The average CES-D scores and the proportions of participants with antidepressant use are shown in Figures A and B, respectively. CES-D: Center for Epidemiological Studies-Depression.

To identify the best trajectory model, we explored 2 polynomial orders (ie, linear and quadratic) with different numbers of trajectory groups in the group-based multi-trajectory modeling. The best model with 3 trajectory groups with a linear trend was chosen based on the model selection criteria (Figure 3). According to the trend across 3 exams, the patterns of the 3 trajectories were classified as “low symptom,” “moderate symptom,” and “high symptom” groups. The low symptom group (570/722, 79%; mean follow-up 287, SD 109 days) consisted of participants who had consistently low CES-D scores (mean CES-D 4.64 at exam 1, 3.72 at exam 2, and 4.06 at exam 3) and more than 95% of them did not report antidepressant medication use across exam 1 to exam 3. The moderate symptom group (71/722, 9.8%; mean follow-up 280, SD 118 days) comprised participants who exhibited intermediate CES-D scores (mean CES-D 6.56 at exam 1, 5.06 at exam 2, and 5.73 at exam 3). About 67.6% (48/71) of participants in the moderate symptom group took antidepressants at exam 1, 87.3% (62/71) at exam 2, and 91.5% (65/71) at exam 3. The high symptom group (81/722, 11.2%; mean follow-up 252, SD 116 days) consisted of participants who had the highest CES-D scores (mean CES-D 17.5 at exam 1, 17.2 at exam 2, and 18 at exam 3), surpassing the threshold CES-D score of 16 consistent with the presence of depressive symptoms. Among participants in the high symptom group, 21% (17/81) at exam 1, 33.3% (27/81) at exam 2, and 46.9% (38/81) at exam 3 reported the use of antidepressants. Both the moderate and high symptom groups increased the antidepressant medication use from exam 1 to exam 3. However, a larger proportion of participants reported antidepressants across exams 1 to 3 in the moderate symptom group than in the high symptom group.

We also observed substantial differences in the other study characteristics (Table 1). Compared to the low symptom group (mean age 52 years, mean BMI 27.9), the participants in the moderate (mean age 55 years, mean BMI 29.6) and high symptom groups (mean age 54 years, mean BMI 29.5) were older and heavier. Among the 3 trajectory groups, the participants in the moderate symptom group were the most likely to be women (57/71, 80.3% compared to 317/579, 55.6% in the low symptom group and 58/81, 71.6% in the high symptom group). In addition, the participants in the high symptom group exhibited the lowest wear hours per day (mean wear hour per day was 12.1 compared to 13.1 hours per day in the low symptom group and 12.8 wear hours per day in the moderate symptom group). The high symptom group also had the shortest follow-up duration (mean follow-up days 252 compared to 287 in the low symptom group and 280 in the moderate symptom group).

**Table 1:** Characteristics of 722 participants by trajectory group^a^.

Characteristics		Low symptom	Moderate symptom	High symptom	*P* value	
Total, n (%)		579 (79)	71 (9.8)	81 (11.2)	N/A^b^	
Age (years), mean (SD)		52 (8.7)	55 (7.7)	54 (7.9)	.02	
Sex (female), n (%)		317 (55.6)	57 (80.3)	58 (71.6)	<.001	
Wear hours per day, mean (SD)		13.1 (2.12)	12.8 (2.07)	12.1 (2.31)	<.001	
Follow-up days, mean (SD)		287 (109)	280 (118)	252 (116)	.03	
BMI, mean (SD)		27.9 (5.28)	29.6 (6.82)	29.5 (6.29)	.004	
Current smoking (yes), n (%)		28 (4.9)	1 (1.4)	7 (8.6)	.12	
Marital status (married), n (%)		443 (77.7)	53 (74.6)	45 (55.6)	<.001	
Hypertension (yes), n (%)		251 (44)	35 (49.3)	48 (59.3)	.03	
**Education, n (%)**						.24
	Less than high school	2 (0.4)	0 (0)	0 (0)		
	Completed high school or some college	162 (28.4)	27 (38)	33 (40.7)		
	Bachelor’s degree or higher	403 (70.7)	44 (62)	48 (59.3)		
Fasting blood glucose, mean (SD)		97.7 (17.0)	98.1 (17.0)	95.5 (13.7)	.52	
Diabetes status, n (%)		29 (5.1)	4 (5.6)	6 (7.4)	.69	
Prevalent CVD^c^, n (%)		24 (4.2)	3 (4.2)	2 (2.5)	.75	
HDL^d^, mean (SD)		61.2 (20.0)	62.7 (18.1)	60.8 (17.8)	.81	
Total cholesterol, mean (SD)		188 (36.8)	199 (36.7)	194 (36.7)	.03	
Triglycerides, mean (SD)		105 (76.6)	111 (46.3)	107 (66.6)	.85	
Lipid treatment		111 (19.5)	22 (31.0)	12 (16.0)	.046	

^a^Wear hour and follow-up days of step counts were measured during 1-year follow-up during eFHS, and all the other variables were measured at exam 3 (Year 2016-2019).

^b^N/A: not applicable.

^c^CVD: cardiovascular disease.

^d^HDL: high-density lipoprotein.

### Association of Depressive Symptom Trajectory Groups With Physical Activity

**Figure 4:**
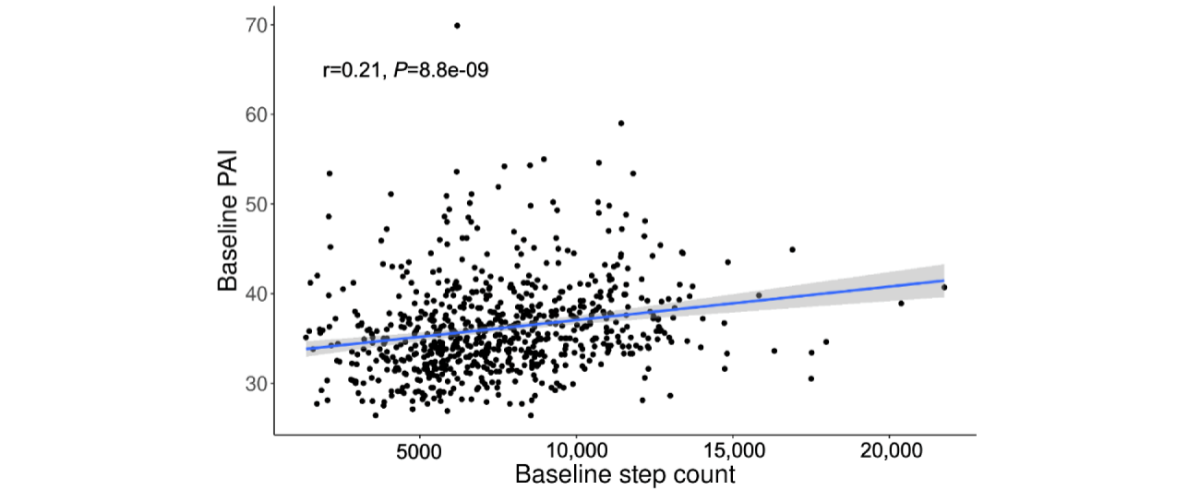
Correlation between the first app-based PAI and step counts during first 30 days in electronic Framingham Heart Study (eFHS). PAI: physical activity index.

We observed a weak positive correlation between the first app-based PAI and smartwatch-collected step counts during the first 30 days (Pearson correlation coefficient *r*=0.21; Figure 4). We further quantified the association between trajectory groups and the first PAI values collected from the eFHS smartphone app (Table 2). Adjusting for age and sex, participants in the high symptom group had, on average, a 0.83 smaller PAI compared to the low symptom group (95% CI –2.05 to 0.38), but the *P* value did not reach statistical significance (*P*=.18). After further adjusting for other covariates, a greater and significant decrease in the PAI for the participants in the high symptom group was observed. They had a 1.29 smaller PAI compared to the low symptom group (95% CI –2.52 to –0.06; *P*=.04). Furthermore, the PAI of participants in the moderate symptom group was 1.08 smaller than that of the low symptom group after we adjusted for all covariates. However, this difference was not statistically significant (*P*=.10).

We also observed a significant association between the trajectory groups and the average daily step counts during the first 30 days collected by smartwatch after adjusting for age, sex, and average daily wear hour (Table 2). On average, the participants in the moderate symptom group walked 945 fewer daily steps (95% CI –1573 to –318; *P*=.003) compared to the low symptom group. After we further adjusted for additional covariates, the association was attenuated: compared to the low symptom trajectory group, the participants in the moderate symptom group walked 733 fewer steps (95% CI –1344 to –121; *P*=.02), which was a 22% decrease in the magnitude of association (Table 2). The high symptom group, compared to the low symptom group, showed a decrease of 340 daily steps (95% CI –931 to 251; *P*=.26) in the base model that adjusted for age, sex, and average daily wear hour, and displayed a decrease of 208 daily steps (95% CI –790 to 373; *P*=.48) in the multivariable-adjusted model, but these differences were not statistically significant.

In the longitudinal study, we assessed the association between trajectory groups and the longitudinal PAI values collected by smartphone app multiple times within 1 year (Table 3). In the base model that adjusted for age, sex, and number of days, the difference in longitudinal PAI comparing the high symptom group to low symptom group was –0.7 and nonsignificant (95% CI –1.77 to 0.37; *P*=.20). After adjusting for relevant covariates, we observed a significant decrease of 1.09 in longitudinal PAI for the high symptom group compared to the low symptom group (95% CI –2.16 to –0.01; *P*=.048). In addition, the difference in the longitudinal PAI between moderate symptom group and low symptom group was not significant (mean difference –0.98, 95% CI –2.11 to 0.15; *P*=.09) after adjusting for all covariates.

Furthermore, we observed that the trajectory groups were significantly associated with the longitudinal daily step counts collected within 1-year of follow-up. The moderate symptom group, on average, had 1080 fewer daily steps than the low symptom group (95% CI –1695 to –465; *P*=.001) after adjusting for sex, age, average daily wear hour, and number of days. Adjusting for additional covariates attenuated the association: participants in the moderate symptom group walked 823 fewer daily steps on average compared to those in the low symptom group (95% CI –1421 to –226; *P*=.007). The high symptom group walked 586 steps fewer than the low symptom group in the longitudinal step count analysis in the base model that adjusted for sex, age, average daily wear hour, and number of days (95% CI –1161 to –10; *P*=.046). However, the difference was attenuated and no longer significant after adjusting for all covariates (mean difference –374, 95% CI –941 to 193; *P*=.20).

**Table 2:** Association between trajectory groups and baseline physical activity outcomes.

Outcome	Model 1^a^					Model 2^b^			
	High symptom vs low symptom, mean difference (95% CI)	*P* value	Moderate symptom vs low symptom, mean difference (95% CI)	*P* value	High symptom vs low symptom, mean difference (95% CI)		*P* value	Moderate symptom vs low symptom, mean difference (95% CI)	*P* value
Physical activity index	–0.83 (–2.05 to 0.38)	.18	–1.09 (–2.40 to 0.21)	.10	–1.29 (–2.52 to –0.06)		.04	–1.08 (–2.38 to 0.22)	.10
Daily step counts	–340 (–931 to 251)	.26	–945 (–1573 to –318)	.003	–208 (–790 to 373)		.48	–733 (–1344 to –121)	.02

^a^Model 1 covariates include sex, age, and daily wear hour (only for outcome daily step counts) at examination 3.

^b^Model 2 covariates include sex, age, daily wear hour (only for outcome daily step counts), BMI, smoking status, prevalent cardiovascular disease, current diabetes status, education, marital status, hypertension status, fasting blood glucose, high-density lipoprotein cholesterol, total cholesterol, triglycerides, and lipid treatment at examination 3.

**Table 3:** Association between trajectory groups and longitudinal physical activity outcomes during the 1-year follow-up.

Outcome	Model 1^a^					Model 2^b^			
	High symptom vs low symptom, mean difference (95% CI)	*P* value	Moderate symptom vs low symptom, mean difference (95% CI)	*P* value	High symptom vs low symptom, mean difference (95% CI)		*P* value	Moderate symptom vs low symptom, mean difference (95% CI)	*P* value
Physical activity index	–0.70 (–1.77 to 0.37)	.20	–1.03 (–2.17 to 0.12)	.08	–1.09 (–2.16 to –0.01)		.048	–0.98 (–2.11 to 0.15)	.09
Daily step counts	–586 (–1161 to –10)	.046	–1080 (–1695 to –465)	.001	–374 (–941 to 193)		.20	–823 (–1421 to –226)	.007

^a^Model 1 covariates at examination 3 include sex and age; covariates during 1-year follow-up include daily wear hour (for outcome daily step counts only) and number of days.

^b^Model 2 covariates at examination 3 include sex, age, daily wear hour (only for outcome daily step counts), BMI, smoking status, prevalent cardiovascular disease, current diabetes status, education, marital status, hypertension status, fasting blood glucose, high-density lipoprotein cholesterol, total cholesterol, triglycerides, lipid treatment, and number of days.

## Discussion

### Principal Findings

In this study, we constructed depressive symptom trajectory groups based on CES-D scores and antidepressant medication use collected from the FHS research exams. We identified 3 distinct trajectory groups using data from the 3 research exams conducted over a 14-year span prior to the initiation of eFHS. We observed that the low symptom group exhibited consistently low CES-D scores, with an extremely low proportion of participants reporting antidepressant medication use across all 3 exams. On the other hand, the moderate symptom group comprised participants with intermediate CES-D scores, who showed the highest and increasing likelihood of reporting antidepressant medications from exam 1 to exam 3. Last, the high symptom group consisted of participants with the highest CES-D scores that met the threshold for the presence of depressive symptoms. The proportion of participants reporting antidepressant medications in this group fell between the other 2 groups and was observed to be increasing across examinations. We further evaluated the associations of depressive symptom trajectory groups with self-reported PAI and objectively measured step counts collected from eFHS. In models adjusting for important confounders, compared to the low symptom group, the participants in the high symptom group had significantly lower self-reported PAI values collected from the eFHS smartphone app at first return and within the 1-year follow-up. In addition, the participants in the moderate symptom group walked significantly fewer average daily steps during the first 30 days and within the 1-year follow-up when compared to the low symptom group after accounting for important covariates. The moderate symptom group reported the highest antidepressant medication use with more than 90% of participants reporting use at the last timepoint. Our finding is consistent with another study reporting an association between antidepressant use and lower levels of physical activity among women with diabetes [26].

This study differs from most of the previous studies in the following 3 ways. First, the participants in eFHS have been followed for 14 years prior to their participation in eFHS. This enabled us to study the trajectory of depressive symptoms and antidepressant medication use over the course of 14 years and 3 examinations prior to the eFHS. In contrast, few previous studies considered longitudinal trends of depression and depressive symptoms including subclinical thresholds. Most previous studies performed an association of physical activity with depression at one time point [27,28]. Some other studies focused on the association between average levels of depression and physical activity in a longitudinal design with depression at a few time points as the predictor [11,29]. Furthermore, this study used both self-reported PAI and objectively measured step counts within the 1-year follow-up in a community sample. These 2 physical activity measures captured different aspects of a participant’s physical activity level, and they each have advantages and disadvantages. The smartwatch-collected step counts data provided a more objective measure of physical activity than self-reported questionnaires that were used in most of the previous studies, which can be affected by many factors, such as social desirability or recall bias. Compared to the questionnaires used in previous studies, our app-based PAI surveys allowed the participants to complete them in a home-based setting, and they were deployed multiple times within the 1-year period. Compared to the step count data, the PAI scores also quantified the amount and intensity of physical activity performed by an individual over a specific period, thus providing a more comprehensive evaluation of an individual’s overall physical activity level [30]. Last, the integration of both CES-D scores and antidepressant medication across 3 research exams in trajectory modeling gave more complete information than using CES-D scores only. The classification of participants in trajectory groups did not depend solely on 1 exam or 1 variable, instead, the classification of participants revealed the long-term underlying patterns across exams and reflected both depressive symptoms and antidepressant medication use.

The main findings in this study were consistent with those from earlier studies, that is, depressive symptoms were associated with lower subsequent physical activity. The Whitehall study, the Coronary Artery Risk Development in Young Adults study, and other studies analyzed the association between depression at baseline and subsequent self-reported physical activity [11-13]. The 1958 British Birth Cohort used the depression phenotype at a few time points as an exposure of interest in a longitudinal setting to analyze the association between the average levels of depression and physical activity [11]. Previous findings also reported that both depressive symptoms and physical activity played an important role in promoting overall cardiovascular health and well-being. A previous study recruited 125 women from cardiac health screening events to investigate depressive symptoms and health-promoting lifestyle behaviors [31]. This study noted that higher levels of depressive symptoms were significantly associated with poorer health behaviors; early detection and treatment of depression were likely to improve quality of life [31]. Previous discoveries in affected science proposed a new theoretical framework called the upward spiral theory of lifestyle change, which explained how positive affect could contribute to long-term adherence to positive health behaviors [32]. A study that involved 204 patients during recovery from myocardial infarction reported that patients with depression after an acute myocardial infarction were more likely to fail to meet the recommended level of lifestyle changes; the failure to adopt a healthy lifestyle such as engaging in regular physical activity, in turn, contributed to a higher risk of cardiovascular events [33].

Our study represents an effective strategy to leverage health variables collected in traditional research exams with health outcomes collected by mHealth devices. First, the use of mHealth makes it possible for the participants to record their daily routines and lifestyles in a home-based setting, which is more representative of their true physiological behaviors. Therefore, mHealth provides the participants with more precise tracking of their health compared to traditional clinical health exams that record health at 1 point in time. Second, some studies reported that mHealth with an intervention on mood could improve physical activity, and subsequently, health outcomes. The BEHOLD-8 Controlled Clinical Trial examined the impact of a novel phone-delivered positive psychology-motivational interviewing intervention to promote physical activity in patients with type 2 diabetes. This study reported that the positive psychology-motivational interviewing intervention was feasible and well-accepted [34]. The Promoting Activity in Cardiac Patients via Text Messages pilot study identified the association between SMS text messaging focused on well-being and improvements in physical activity and mental health among 40 patients with prior acute coronary syndrome [35]. Therefore, the findings from our study and the previous studies suggest that mood interventions may be essential for mHealth studies that are designed to promote PA levels. Future mHealth studies to promote PA should intervene on mood through such strategies as improving sleep, counseling (well-based), supportive messaging, and activity promotion. Physical activity is a cornerstone of CVD prevention yet nearly half of Americans fail to meet physical activity guidelines. Addressing mood may hold promise to improve engagement in healthy behaviors such as increasing physical activity.

This study had several limitations. First, the sample size was substantively reduced from 1186 to 722 after applying the 3 exclusion criteria that were related to adherence. Low adherence has been a critical limitation in most mHealth studies [36,37]. Despite reduced adherence during the follow-up, the eFHS participants had higher adherence to smartwatch use compared to most mHealth studies [38]. In addition, this study was an observational study, and the causal relationship between depressive symptoms and physical activity cannot be determined although the depressive symptom trajectory groups were constructed prior to physical activity data. Furthermore, several previous studies have shown that physical activity and depressive symptoms have bidirectional associations. That is, people with depressive symptoms were more likely to have a lower level of physical activity at follow-up, and the lack of physical activity can predict an increased likelihood of depressive symptoms at follow-up. However, since we do not have CES-D scores collected in the health research center after mHealth physical activity, we are unable to investigate whether physical activity predicts CES-D scores at the next exam. Last, the eFHS participants were mostly White, middle-aged, with higher education, resided in New England, and were healthier than all participants attending examinations (Multimedia Appendix 1). Therefore, the generalization of the findings in this study to individuals in other age ranges, races or ethnicities, and different geographical areas may be limited.

### Conclusions

We observed that antecedent depressive symptom trajectories were associated with subsequent physical activity collected in eFHS. Compared to the low symptom group with consistently low CES-D scores and proportions of antidepressant use, the participants in the high symptom group with high CES-D scores and increasing likelihood of taking antidepressants had decreased levels of app-based PAI values. In addition, the participants in the moderate symptom group with intermediate CES-D scores and the highest reported antidepressant medication use walked fewer average daily step counts. Further exploration is needed to explore interventions aimed at enhancing mood, specifically through the use of mHealth technologies, with the goal of encouraging individuals to engage in regular physical activity.

## Appendix

Multimedia Appendix 1 Comparison between included participants in our analysis and excluded participants.

## Abbreviations

CES-D: Center for Epidemiological Studies-Depression
CVD: cardiovascular disease
eFHS: electronic Framingham Heart Study
FHS: Framingham Heart Study
Gen 3: third generation
mHealth: mobile health
NOS: New Offspring Spouse
PAI: physical activity index
STROBE: Strengthening the Reporting of Observational Studies in Epidemiology
