# Supplementary material for: Association of Depressive Symptom Trajectory With Physical Activity Collected by mHealth Devices in the Electronic Framingham Heart Study: Cohort Study
Source: JMIR Ment Health. 2023 Jul 14;10:e44529. doi: 10.2196/44529 (PMC10382951; doi:10.2196/44529)
Supplement: Multimedia Appendix 1 [file mental_v10i1e44529_app1.docx]

**Multimedia Appendix 1.** Comparison between included participants in our analysis and excluded participants.

| **Group** | | 1 | 2 | 3 |  |  |
| --- | --- | --- | --- | --- | --- | --- |
| **Variables at exam 3** | | FHS^a^ participants at research exam 3 not enrolled in eFHS | Other eFHS^b^ participants who didn’t meet inclusion criteria | Study sample: eFHS participants with mobile step data | *P* value: compare group 1 versus 3 | *P* value: compare group 2 versus 3 |
| Sample Size | | 1275 | 1255 | 722 |  |  |
| Age (years), mean (SD) | | 57(10) | 53 (9) | 53 (8.5) | <.01 | .50 |
| Sex female, n (%) | | 632 (50) | 670 (53) | 432 (60) | <.01 | <.01 |
| BMI (kg/m^2^), mean (SD) | | 28.9 (6) | 28.4 (5.7) | 28.2 (5.6) | .04 | .74 |
| Physical Activity Index score, mean (SD) | | 34.5 (6.3) | 33.6 (5.3) | 33.6 (4.8) | <.01 | .98 |
| **Education, n (%)** | | | | | | |
|  | Less than high school | 28 (2) | 12 (1) | 2 (0.3) | <.01 | .14 |
|  | Completed high school or some college | 625 (49) | 435 (35) | 222 (31) | <.01 | .09 |
|  | Bachelor’s degree or higher | 605 (47) | 802 (64) | 495 (69) | <.01 | .04 |
| CES-D score, mean (SD)^c^ | | 7.27 (7.47) | 6.34 (6.83) | 5.79 (6.63) | <.01 | .08 |
| **Blood Pressure at research exam 3** | | | | | | |
|  | Systolic, mean (SD) | 122 (15) | 119 (14) | 118 (14) | <.01 | .02 |
|  | Diastolic, mean (SD) | 76 (9) | 76 (8) | 75 (9) | .44 | .20 |
| Current Smoking, n (%) | | 94 (7) | 73 (6) | 36 (5) | .05 | .49 |
| Alcohol Consumption (average drinks per week), mean (SD) | | 5 (8) | 5 (7) | 5 (6) | .31 | .02 |
| Married, living as married, or living with partner, n (%) | | 832 (65) | 909 (72) | 541 (75) | <.01 | .26 |
| Antihypertensive Use, n (%) | | 389 (31) | 261 (21) | 153 (21) | <.01 | .91 |
| Antidepressant Use, n (%) | | 206 (16) | 210 (17) | 127 (18) | .46 | .69 |

^a^FHS: Framingham Heart Study.

^b^eFHS: electronic Framingham Heart Study.

^c^CES-D: center for epidemiological studies-depression.
